# Hereditary Angioedema with Normal C1-Inhibitor in Children: Diagnostic Challenges, Novel Mutations in *MYOF*, *F12*, and *HS3ST6*, and the Role of Bioinformatics Analysis

**DOI:** 10.3390/ijms27219551

**Published:** 2026-10-26

**Authors:** Anita R. Denisova, Natalia G. Kolosova, Natalia A. Geppe, Veronika D. Denisova, Vera A. Podzolkova, Irina V. Ozerskaia, Svetlana I. Shatalina, Marina D. Shakhnazarova, Yulia V. Ostankova, Nadezhda A. Pechnikova

**Affiliations:** 1 Department of Children’s Diseases, N.F. Filatov Clinical Institute of Children’s Health, Sechenov First Moscow State Medical University, Moscow 119435, Russia; 2 Department of Propaedeutics of Children’s Diseases, N.F. Filatov Clinical Institute of Children’s Health, Sechenov First Moscow State Medical University, Moscow 119435, Russia; 3 Research Clinical Institute of Childhood of the Ministry of Health of the Moscow Region, Moscow 141009, Russia; 4 Saint Petersburg Pasteur Institute, 14 Mira St., Saint Petersburg 197101, Russia; shenna1@yandex.ru; 5 Elpida BioPharm P.C., 54636 Thessaloniki, Greece; 6 Laboratory of Chemical Engineering A’, Department of Chemical Engineering, Faculty of Engineering, Aristotle University of Thessaloniki, 54636 Thessaloniki, Greece

**Keywords:** hereditary angioedema with normal C1 inhibitor, *MYOF*, *F12*, *HS3ST6*, bioinformatics

## Abstract

Hereditary angioedema with normal C1 inhibitor (HAE-nC1INH) lacks validated biochemical biomarkers, complicating diagnosis and interpretation of rare genetic variants, particularly in children. To improve molecular characterization of pediatric HAE-nC1INH, we studied 23 children with recurrent non-histaminergic angioedema, normal C4 and C1 inhibitor levels, and no pathogenic *SERPING1* variants. All 23 patients underwent whole-exome sequencing, followed by integrated in silico assessment of candidate variants. A rare heterozygous *MYOF* p.Arg1590Gln variant was identified in one patient and classified as a VUS, with computational analyses suggesting potential functional relevance. Homozygous *F12* p.Ala207Pro was identified in two unrelated patients; however, its high population frequency and benign ClinVar classification do not support a causative role. Four *HS3ST6* missense variants were detected in five patients; p.Pro199Leu showed the strongest computational evidence of potential functional relevance, whereas the clinical significance of the remaining variants remains uncertain. These findings expand the molecular spectrum of pediatric HAE-nC1INH and support combining sequencing with prioritization of variants in relevant genes. Functional studies are required to confirm causality before clinical implementation.

## 1. Introduction

Hereditary angioedema (HAE) is a rare genetic disorder characterized by recurrent, typically self-limiting episodes of localized swelling involving the skin and subcutaneous tissues, gastrointestinal tract and upper airways [1]. A systematic review and meta-analysis of 24 studies comprising 11,245 diagnosed cases estimated a pooled global prevalence of 1.22 cases per 100,000 individuals, although regional differences in diagnostic access and patient registration probably lead to underestimation [2]. Despite its rarity, HAE imposes a substantial clinical and humanistic burden, including pain, disruption of daily activities, reduced work productivity and impaired quality of life [3,4]. Laryngeal oedema represents the most serious manifestation because progressive upper-airway obstruction can result in fatal asphyxiation in the absence of timely disease-specific treatment [1,3,5].

In the best-characterized forms of HAE, swelling results from excessive bradykinin generation, activation of bradykinin B2 receptors on endothelial cells and a consequent increase in vascular permeability [1,5,6]. This mechanism distinguishes HAE from mast-cell-mediated angioedema, in which histamine and other mast-cell mediators predominate. Antihistamines, systemic glucocorticoids and epinephrine are therefore ineffective in confirmed bradykinin-mediated attacks. Elucidation of the kallikrein–kinin pathway has enabled the development of targeted therapies based on C1 inhibitor replacement, inhibition of plasma kallikrein or activated FXII, and antagonism of the bradykinin B2 receptor [1,5].

Most cases of HAE are caused by pathogenic variants in *SERPING1*, which result either in a quantitative deficiency of C1 inhibitor (HAE-1) or reduced functional activity despite normal or increased antigenic concentrations (HAE-2) [1,5]. C1 inhibitor regulates several interconnected proteolytic cascades, including the complement, contact, coagulation and fibrinolytic systems. Loss of C1 inhibitor activity permits dysregulated activation of FXII and plasma kallikrein, leading to excessive bradykinin release [1,5,6]. The molecular basis of HAE-1 and HAE-2 is comparatively well established, and laboratory confirmation relies on measurements of C4 and the antigenic concentration and functional activity of C1 inhibitor [1,5].

A distinct clinical and genetic group comprises patients with recurrent angioedema despite normal C1 inhibitor concentration and function. Previously termed HAE type III (HAE-3), this condition is now designated HAE with normal C1 inhibitor (HAE-nC1INH) [7,8,9]. Diagnosis remains challenging because routine complement testing is typically unremarkable and no universally validated, sensitive and specific laboratory biomarker is available [7,8]. Diagnostic evaluation therefore requires integration of the clinical phenotype and family history, exclusion of mast-cell-mediated, drug-induced and acquired angioedema, and molecular genetic testing [7,8,9]. Incomplete penetrance, variable expressivity and limited evidence concerning the mechanisms and treatment responses of individual subtypes create additional uncertainty [7,9].

Molecular studies have demonstrated substantial genetic heterogeneity in HAE-nC1INH. Genetically defined subtypes have been associated with pathogenic or putatively pathogenic variants in *F12*, *PLG*, *ANGPT1*, *KNG1*, *MYOF*, and *HS3ST6* [6,7,8,9] while variants in *CPN1* and *DAB2IP* have more recently been implicated in additional angioedema phenotypes [10,11,12]. However, the level of evidence supporting individual genes varies considerably. Several gene–disease associations are based on single families, small cohorts, or limited functional validation, and no pathogenic variant in a recognized HAE-associated gene can be identified in a substantial proportion of patients with a convincing clinical phenotype [8,9]. These observations indicate that the genetic architecture of HAE-nC1INH remains incompletely understood and suggest that additional genes, regulatory variants, and oligogenic or multifactorial mechanisms contributing to endothelial dysfunction and vascular permeability remain to be identified.

Among genetically defined forms, HAE-FXII is the best-characterized subtype. Most pathogenic variants affect Thr328 in the FXII (Thr309 in the mature protein), disrupting O-linked glycosylation and increasing susceptibility to proteolytic activation, thereby enhancing bradykinin generation through the contact activation pathway [6,13]. In contrast, the pathogenic roles of mutations in *MYOF* and *HS3ST6* remain less well established. MYOF was initially implicated through the identification of a gain-of-function variant in a single family, and subsequent studies have reported additional rare variants in unrelated patients; however, independent replication and functional validation remain limited [14,15,16]. Likewise, the *HS3ST6* variant c.430A>T (p.Thr144Ser) was first identified in three affected members of a multigenerational family and was associated with impaired heparan sulfate 3-O-sulfation [17]. More recent whole-exome sequencing (WES) studies have expanded the spectrum of *MYOF* and *HS3ST6* variants but have also highlighted considerable genetic and phenotypic heterogeneity, as well as variable therapeutic responses among affected individuals [16].

The diagnosis of HAE during childhood presents additional challenges. Peripheral swelling and recurrent abdominal pain are non-specific and may be misattributed to allergic, gastrointestinal or surgical conditions [18,19]. In HAE-nC1INH, diagnostic uncertainty is compounded by normal routine complement results and limited information on the age-dependent penetrance and natural history of individual genetic subtypes [7,19]. Most published paediatric evidence concerns HAE caused by C1 inhibitor deficiency, whereas the clinical spectrum and genetic diversity of paediatric HAE-nC1INH remain poorly characterized [18,19]. Consequently, affected children may undergo repeated investigations and hospitalizations and receive treatments that do not target the underlying disease mechanism.

The genetic heterogeneity of HAE-nC1INH supports the use of next-generation sequencing, including WES, to interrogate the coding regions of multiple genes and identify known and previously unreported rare variants [16,20,21]. However, rarity alone does not establish pathogenicity. Variant interpretation should integrate population frequency, the proposed mode of inheritance, intrafamilial segregation, phenotypic specificity, published functional evidence and calibrated computational predictions [20,22,23]. Analyses of evolutionary conservation, protein structure and stability, molecular interactions and splicing can support variant prioritization, but remain primarily supporting and hypothesis-generating evidence rather than substitutes for experimental validation [22,23]. Furthermore, a negative exome result does not exclude a genetic etiology, because causal changes may lie outside captured coding regions, involve structural variation or remain undetected owing to technical and bioinformatic limitations [20,24].

Despite the expanding spectrum of genes associated with HAE-nC1INH, the pathogenic significance of many rare variants remains unresolved and genotype–phenotype relationships are incompletely defined. These limitations are particularly pronounced in paediatric patients and for variants in *MYOF* and *HS3ST6*, for which specific biomarkers and reproducible functional models are lacking. Here, we investigated the molecular genetic basis of HAE-nC1INH in a paediatric cohort using WES and an integrated bioinformatic framework. We focused on the identification and characterization of rare variants in *MYOF*, *F12* and *HS3ST6*, assessment of their predicted structural and functional consequences, and evaluation of their potential contribution to the observed clinical phenotype.

## 2. Results

### 2.1. Demographic and Clinical Characteristics of the Cohort

All 23 patients underwent initial *SERPING1* testing followed by WES. No pathogenic *SERPING1* variants were identified, and no separate targeted HAE-associated gene panel was performed. WES identified the heterozygous *MYOF* p.Arg1590Gln variant in one patient, homozygous *F12* p.Ala207Pro in two unrelated patients, and four *HS3ST6* missense variants in five patients. Overall, variants prioritized for further evaluation were identified in 8 of 23 patients (34.8%). In the remaining 15 patients (65.2%), no candidate variant considered relevant to the present analysis was identified. This finding does not exclude genetic contributions involving variants or mechanisms not detectable or interpretable by the analytical approach used in this study and warrants further investigation. Females accounted for 14 of 23 patients (60.9%), and the median age at evaluation was 8 years (range, 2–14 years) (Table 1). A positive family history of angioedema was documented in 8 patients (34.8%). In one family (4.3% of the cohort), a relative had died from laryngeal edema, illustrating the potential severity of upper airway involvement.

Available family data were insufficient for informative segregation analysis. In the family of the patient carrying *MYOF* p.Arg1590Gln, the mother had recurrent abdominal attacks and the grandmother had a history of laryngeal edema; however, neither relative was genotyped for p.Arg1590Gln, precluding assessment of cosegregation. In one family carrying *F12* p.Ala207Pro, the index patient was homozygous and the mother was heterozygous. These findings were insufficient to establish cosegregation between the variant and the clinical phenotype. No informative segregation data were available for the second *F12* family. Accordingly, segregation-based evidence was not used to support the pathogenicity of either variant. The first clinical manifestations occurred within the first decade of life in 16 patients (69.6%). Age at disease onset ranged from 1 to 11 years, with a median of 6 years. At disease onset, subcutaneous angioedema was observed in 13 patients (56.5%). Abdominal attacks were reported in 10 patients (43.5%), including isolated abdominal attacks without concomitant edema in 7 patients (30.4%) and abdominal pain accompanied by peripheral edema in 3 patients (13.0%). In one child (4.3%), the first episode involved upper airway edema requiring emergency medical care. Because clinical manifestations at disease onset could overlap within individual patients, these categories were not mutually exclusive (Table 1). When the overall clinical course was considered, recurrent peripheral angioedema was the most frequent manifestation, occurring in 14 patients (60.9%). Abdominal attacks, characterized by paroxysmal abdominal pain without evidence of acute surgical pathology, occurred in 12 patients (52.2%); 5 of these patients also experienced edema at other anatomical sites. Notably, 2 patients (8.7%) underwent unnecessary surgical interventions after abdominal attacks were initially interpreted as acute abdominal pathology. Edema involving the soft tissues of the face and neck was documented in 3 patients (13.0%), whereas isolated limb edema occurred in 2 children (8.7%). Transient episodes of marginated erythema were reported in another 2 patients (8.7%). Collectively, these observations demonstrate substantial heterogeneity in the clinical presentation of pediatric HAE-nC1INH and underscore the diagnostic challenge posed by abdominal and other less typical manifestations.

Population-frequency and database annotations for all six variants identified and evaluated in this study are summarized in Table 2. Population allele frequencies were obtained from the NHLBI TOPMed dataset displayed in NCBI dbSNP RefSNP records (dbSNP Build 157; accessed 11 September 2026). Global, multi-ancestry frequencies are reported for the specific alleles corresponding to each variant. Transcript-level HGVS nomenclature is provided using the corresponding RefSeq transcript accession and version, whereas genomic HGVS descriptions are reported relative to GRCh37/hg19.

### 2.2. Identification and In Silico Characterization of a Novel MYOF Variant

**Figure 1:**
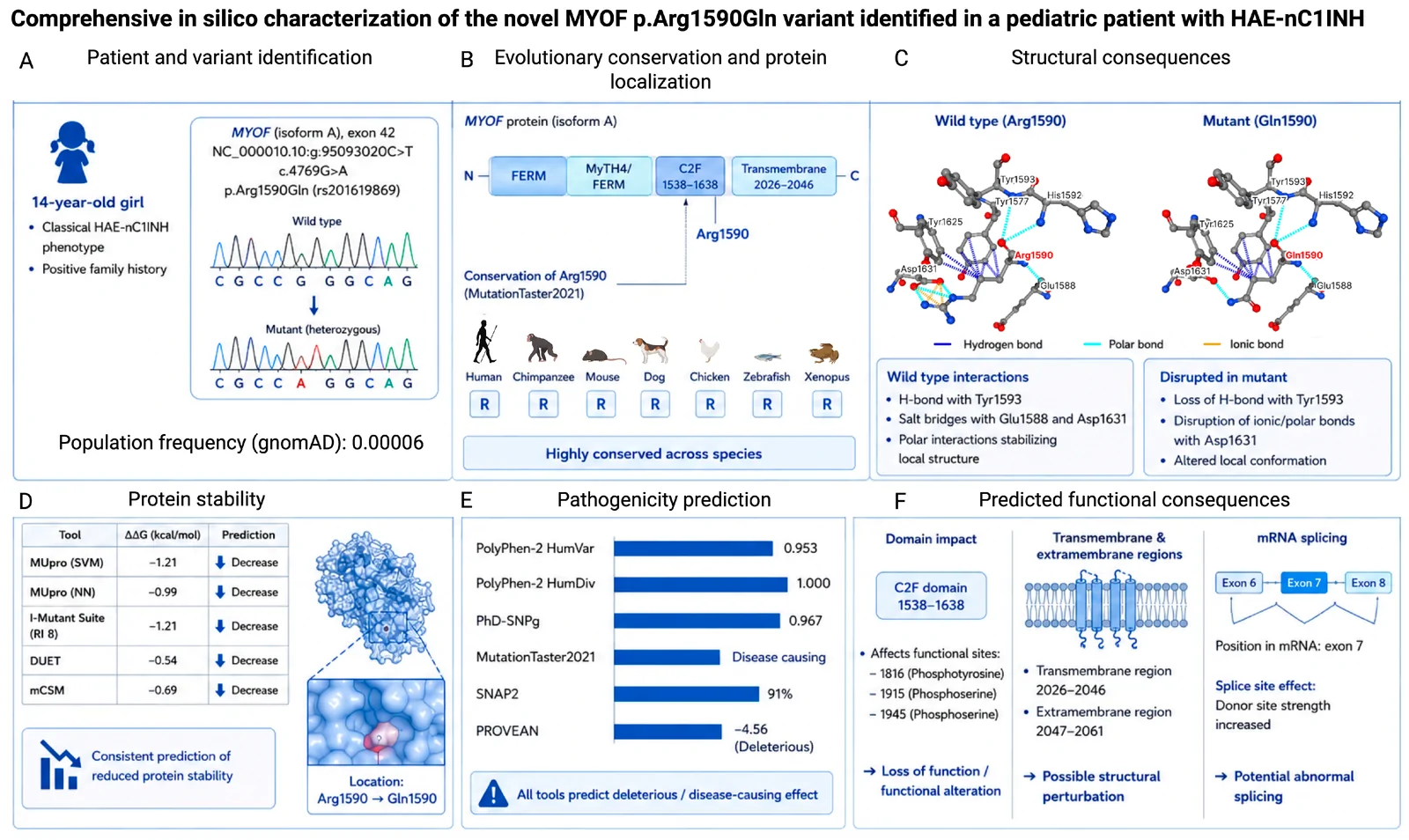
Integrated in silico characterization of the novel *MYOF* p.Arg1590Gln variant identified in a pediatric patient with HAE-nC1INH. Comprehensive computational characterization of the heterozygous *MYOF* p.Arg1590Gln (c.4769G>A; NC_000010.10:g.95093020C>T) variant identified by WES in a 14-year-old girl with a classical HAE-nC1INH phenotype. The workflow summarizes (**A**) clinical presentation and molecular identification of the variant; (**B**) localization of Arg1590 within the highly conserved C2F domain of myoferlin and evolutionary conservation across vertebrate species; (**C**) predicted structural alterations in hydrogen-bonding and ionic interactions following substitution of Arg1590 by glutamine; (**D**) predicted reduction in protein stability according to multiple computational algorithms (MUpro, I-Mutant2.0, DUET, and mCSM); (**E**) computational pathogenicity assessment using complementary prediction tools, including PolyPhen-2, PhD-SNPg, MutationTaster2021, SNAP2, and PROVEAN; and (**F**) predicted functional consequences involving protein structure, membrane-associated domains, and pre-mRNA splicing. Collectively, these in silico findings suggest potential structural and functional effects of the *MYOF* p.Arg1590Gln variant; however, its biological and pathogenic significance requires experimental validation. Symbols and colors: In panel (**A**), Sanger sequencing chromatograms use the conventional nucleotide color code (A, green; C, blue; G, black; T, red). In panel (**B**), “R” denotes arginine conserved at position 1590 across the indicated species, and N and C indicate the amino- and carboxy-termini of the protein, respectively. In panel (**C**), atoms are colored by element (carbon, gray; oxygen, red; nitrogen, blue); blue, cyan and gold dashed lines denote hydrogen, polar and ionic interactions, respectively. In panel (**D**), downward arrows indicate a predicted decrease in protein stability (negative ΔΔG). In panel (**F**), arrows indicate the predicted direction of downstream functional consequences.

WES performed in a 14-year-old girl presenting with recurrent peripheral angioedema involving the hands (1–2 attacks per month), recurrent abdominal attacks requiring repeated hospitalization for suspected acute abdomen, and a positive family history of HAE identified a previously unreported heterozygous missense variant in *MYOF* (isoform A; NC_000010.10:g.95093020C>T, p.Arg1590Gln) (Table 3). The family history included laryngeal edema in the patient’s grandmother and recurrent abdominal attacks in her mother. Conventional treatment with antihistamines and systemic glucocorticoids had been ineffective, supporting a bradykinin-mediated disease mechanism. The identified variant was extremely rare in population databases, with a minor allele frequency of 6 × 10^−5^ (Figure 1A). Because *MYOF* has only recently been implicated as a candidate gene for HAE with HAE-nC1INH, comprehensive in silico analyses were performed to evaluate the potential functional significance of the identified variant. Amino acid conservation analysis demonstrated that Arg1590 is a highly conserved residue across vertebrate species. Substitution of the positively charged arginine with glutamine was predicted to reduce residue size and alter local physicochemical properties within the C2F domain of myoferlin.

Structural modeling predicted disruption of the local interaction network, including loss of hydrogen bonding with Tyr1593 and disruption of ionic interactions involving Asp1631 and Glu1588. Protein stability analysis consistently predicted destabilization of the mutant protein, with MUpro and I-Mutant2.0 both estimating a decrease in Gibbs free energy of approximately ΔΔG = −1.21 kcal/mol, while I-Mutant2.0 assigned a reliability index (RI) of 8, indicating high confidence in the prediction. Multiple computational algorithms consistently supported the deleterious nature of the variant. PolyPhen-2 classified p.Arg1590Gln as probably damaging (HumVar score = 0.953; HumDiv score = 1.000), PhD-SNPg predicted pathogenicity with a probability of 0.967, and MutationTaster2021 predicted disruption of the C2F functional domain, altered splice-site strength, and changes affecting predicted phosphorylation sites and transmembrane architecture (Figure 1B–F). Collectively, the in silico analyses consistently supported the functional relevance of the p.Arg1590Gln variant.

According to ACMG/AMP criteria, the *MYOF* p.Arg1590Gln variant was classified as a VUS, with PP3 (Supporting) based on concordant computational predictions; no strong or moderate pathogenic criteria were met. Criteria designated as “not met” were evaluated but lacked sufficient supporting evidence.

### 2.3. Identification and In Silico Characterization of F12 Variant

**Figure 2:**
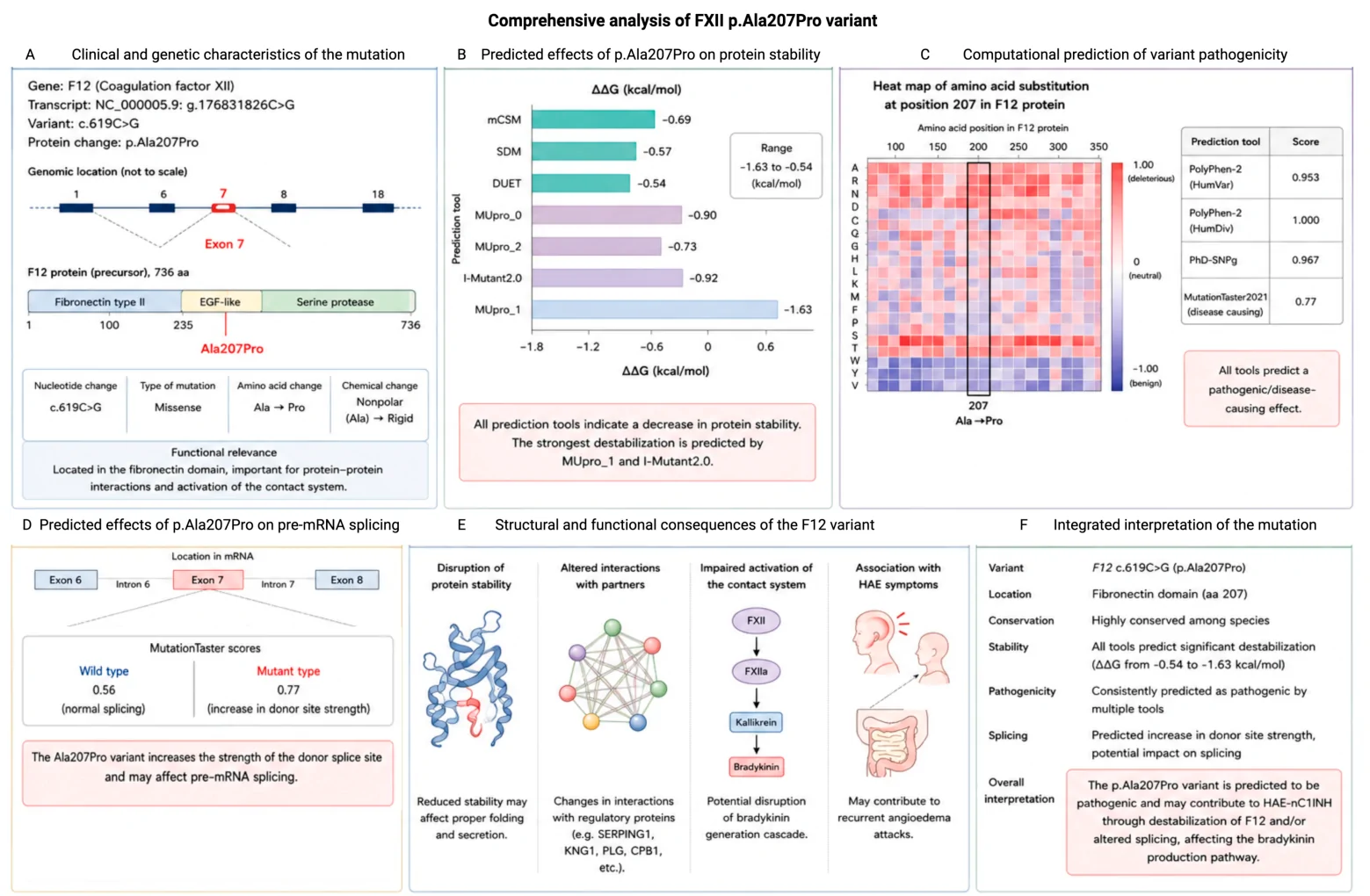
Comprehensive in silico characterization of *F12* p.Ala207Pro variant. (**A**) Clinical and genetic characteristics of the identified *F12* variant, including genomic localization, affected exon, protein domain architecture, and amino acid substitution. (**B**) Predicted effects of p.Ala207Pro on protein stability estimated using multiple computational algorithms (MUpro, I-Mutant2.0, DUET, SDM, and mCSM), with concordant predictions of reduced protein stability. (**C**) Computational pathogenicity assessment using complementary prediction tools, including PolyPhen-2 (HumVar and HumDiv), PhD-SNPg, and MutationTaster2021. (**D**) Predicted effects of p.Ala207Pro on pre-mRNA splicing, including increased donor splice-site strength and a potential influence on alternative splicing. (**E**) Putative structural and functional consequences of the variant, including predicted changes in protein stability and molecular interactions and a potential influence on FXII activation and bradykinin generation. (**F**) Integrated interpretation of structural, stability, splicing, and pathogenicity predictions, suggesting potential functional relevance of p.Ala207Pro in HAE-nC1INH. These computational predictions are discordant and do not provide evidence that p.Ala207Pro contributes to HAE-nC1INH; variant-specific functional studies would be required to establish any biological effect. Color coding: In panel (**A**), blue denotes the reference genomic/protein structure, whereas red highlights exon 7 and the p.Ala207Pro variant. In panel (**B**), negative ΔΔG values indicate predicted destabilization; bar colors distinguish the individual prediction tools and do not represent different biological categories. In panel (**C**), the heat map ranges from blue (benign/neutral substitutions) through white (intermediate effect) to red (deleterious substitutions), as indicated by the scale; the black box highlights amino-acid position 207. In panel (**D**), blue denotes the wild-type sequence/splicing prediction and red denotes the mutant sequence and predicted increase in donor splice-site strength. In panel (**E**), red highlights the altered F12 region and bradykinin, whereas the remaining colors are used schematically to distinguish pathway components and interacting proteins. In panel (**F**), the pale red box highlights the integrated pathogenic interpretation.

A homozygous missense variant in *F12* (NC_000005.9:g.176831826C>G; rs17876030; p.Ala207Pro) (Table 2) was identified in two unrelated patients, a 13-year-old girl and a 2-year-old boy, both presenting with recurrent angioedema predominantly affecting the lips and tongue, intermittent abdominal pain, gastrointestinal manifestations, and chronic inflammatory changes in the oral mucosa. In one family, segregation analysis demonstrated that the patient’s mother carried the variant in the heterozygous state. Comprehensive in silico analysis was performed to evaluate the potential functional consequences of the identified variant. The Ala207 residue is located within the EGF-like 2 domain, a functionally important region involved in FXII activation and positioned adjacent to evolutionarily conserved residues. Substitution of alanine with proline was predicted to increase residue size and molecular volume while altering hydrophobicity, suggesting disruption of local protein conformation. Structural modelling using PremPS predicted loss of a polar interaction with Val187, accompanied by the formation of novel ionic interactions and rearrangement of local molecular contacts. Protein stability analyses performed with multiple computational algorithms consistently predicted destabilization of the mutant protein, with estimated ΔΔG values ranging from −0.54 to −1.63 kcal/mol. Splice-site analysis using MutationTaster2021 predicted strengthening of the donor splice site, with the donor score increasing from 0.56 to 0.77, suggesting a potential effect on alternative splicing and the generation of truncated or functionally altered FXII protein isoforms. Predictions of pathogenicity were partially discordant across computational algorithms. I-Mutant Disease and HOPE supported a deleterious effect of the variant, whereas SNAP2 and PROVEAN predicted a neutral functional impact (Figure 2A–F). Nevertheless, the combined structural, thermodynamic, and splicing analyses indicate that p.Ala207Pro may have functional consequences that warrant further experimental validation and clinical investigation.

However, predictions of functional impact were discordant across the computational algorithms used. Importantly, no functional assays of FXII activation or downstream contact-system activity were performed in the present study. Therefore, these computational findings should be regarded as hypothesis-generating and do not establish that p.Ala207Pro results in enhanced FXII activation or contributes causally to HAE-nC1INH. Functional studies evaluating FXII activation and contact-system activity will be required to determine the biological significance of this variant.

*F12* p.Ala207Pro variant (NM_000505.4:c.619G>C; rs17876030) is common in the general population and is classified as benign in ClinVar, including in the context of HAE type 3. Although in silico predictions are discordant, with some algorithms suggesting potential structural or functional effects, computational evidence alone is insufficient to outweigh the population-frequency and clinical-classification evidence. Accordingly, p.Ala207Pro was not considered a causative variant for HAE-nC1INH in the present study. Its detection in affected individuals is therefore reported descriptively and should not be interpreted as evidence of disease causality. A possible functional or modifier effect cannot be excluded but remains speculative in the absence of supporting functional and informative segregation data. The population-frequency data, ClinVar classification, segregation findings, computational predictions, applicable ACMG/AMP criteria, and final interpretation of p.Ala207Pro are summarized in Table 4.

### 2.4. Identification and In Silico Characterization of HS3ST6 Variants

**Figure 3:**
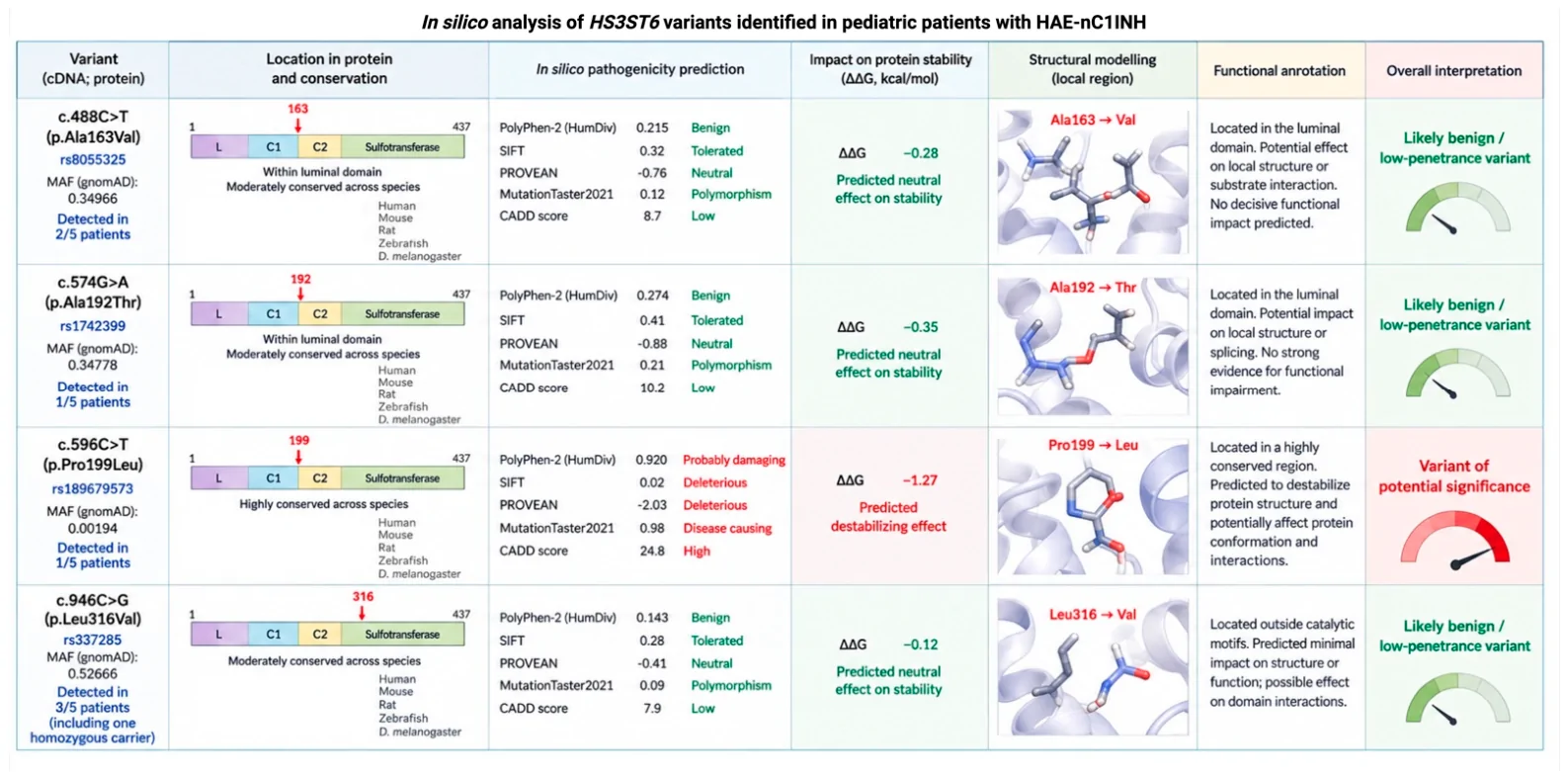
Integrated computational characterization of *HS3ST6* variants identified in HAE-nC1INH. Five unrelated patients carried four different *HS3ST6* missense variants identified by WES. The figure summarizes variant distribution, population frequency, evolutionary conservation, predicted effects on protein stability, predicted structural consequences, and computational functional interpretation. Among the identified variants, p.Pro199Leu showed the strongest in silico evidence of potential functional relevance, including localization within a highly conserved region, predicted protein destabilization, and concordant predictions of a deleterious effect across multiple computational algorithms. Color coding: In the protein-domain schematics, purple, blue, yellow and green denote the luminal (L), C1, C2 and sulfotransferase domains, respectively; red arrows and residue numbers indicate the positions of the identified variants. In the pathogenicity and stability analyses, green denotes benign/tolerated/neutral or low-impact predictions, whereas red denotes damaging/deleterious, disease-causing or destabilizing predictions. In the structural models, carbon atoms are shown in gray, oxygen in red and nitrogen in blue. In the overall interpretation column, green indicates likely benign/low-penetrance variants and red indicates a variant of potential significance. Blue text is used to highlight dbSNP identifiers and variant occurrence in the patient cohort.

Five unrelated patients (one girl and four boys) carried four different missense variants in *HS3ST6*, encoding heparan sulfate 3-O-sulfotransferase 6, an enzyme involved in the 3-O-sulfation of heparan sulfate. The most frequently identified variant was p.Leu316Val (rs337285), which was detected in 3 patients, including one homozygous carrier. Two patients harboured combinations of variants (p.Ala163Val/p.Leu316Val and p.Ala192Thr/p.Ala163Val), whereas one patient carried the rare p.Pro199Leu (rs189679573) variant, with a minor allele frequency of 0.001111 in TOPMed Global (Figure 3).

Comprehensive in silico analysis demonstrated marked differences in the predicted functional consequences of the identified variants. Among them, p.Pro199Leu showed the strongest evidence of functional significance. The substituted residue was located within a highly conserved functional domain of *HS3ST6* and was consistently predicted to be deleterious by multiple independent algorithms, including PolyPhen-2 (HumDiv score = 0.920, prediction score = 98), PROVEAN, and MutationTaster2021. Protein stability analysis further predicted destabilization of the mutant protein, with an estimated ΔΔG of −1.27 kcal/mol (Figure 3). In contrast, p.Ala163Val, p.Ala192Thr, and p.Leu316Val were predicted to have neutral or only modest functional effects. Although structural and functional annotation suggested possible effects on HS3ST6-related molecular interactions, the available computational and population-level data are insufficient to establish a functional or disease-modifying role for these variants. Accordingly, they are considered here as candidate susceptibility variants or putative modifiers whose potential contribution to HAE-nC1INH remains hypothetical and requires further genetic and functional validation. Among the *HS3ST6* variants identified in this cohort, p.Pro199Leu showed the strongest computational evidence of potential functional relevance.

## 3. Discussion

### 3.1. Diagnostic Challenges of HAE-nC1INH in Children

HAE-nC1INH remains one of the most diagnostically challenging forms of hereditary angioedema, particularly in pediatric patients. A clinical phenotype refractory to antihistamines and systemic glucocorticoids may raise suspicion of a bradykinin-mediated mechanism; however, treatment non-response alone does not provide definitive evidence of bradykinin-mediated angioedema [8,9]. Unlike classical HAE caused by SERPING1 deficiency, HAE-nC1INH is characterized by normal C4 concentrations and preserved quantitative and functional C1-INH, making routine laboratory investigations insufficient for establishing the diagnosis [8,9]. Consequently, diagnosis relies on careful clinical phenotyping combined with comprehensive molecular genetic testing. Recent international consensus recommendations and the 2025 WAO guidelines emphasize that genetic analysis has become the principal diagnostic biomarker for genetically defined forms of HAE-nC1INH, although many patients still remain without an identifiable molecular cause [1].

The present cohort reflects these diagnostic challenges. All patients exhibited recurrent non-histaminergic angioedema despite normal complement parameters and preserved C1-INH function, while conventional treatment with antihistamines and systemic glucocorticoids was ineffective. This clinical pattern supports a non-histaminergic mechanism but does not, by itself, establish bradykinin-mediated angioedema. In particular, *MYOF*- and *HS3ST6*-associated forms are thought to involve abnormalities of vascular endothelial function rather than a clearly demonstrated bradykinin-dependent mechanism. These findings support early molecular genetic testing in children with recurrent angioedema, abdominal attacks, or laryngeal edema when mast-cell-mediated and other alternative causes have been excluded.

An equally important challenge lies in the interpretation of sequence variants identified in patients with HAE-nC1INH such as *F12* p.Ala207Pro. Given the genetic heterogeneity of the disorder and the variable strength of evidence supporting individual gene–disease and variant–disease relationships, we adopted a tiered interpretation framework distinguishing established pathogenic variants, previously reported variants supported by clinical and/or functional evidence, candidate variants and VUS, and possible susceptibility or modifier variants. Computational evidence was used only as supporting evidence for variant prioritization and was interpreted together with population frequency, segregation, clinical phenotype, published evidence, and, where available, functional data in accordance with ACMG/AMP principles. Concordance among multiple in silico tools may strengthen the rationale for further investigation but does not independently establish pathogenicity or causality.

### 3.2. MYOF p.Arg1590Gln as a Candidate VUS

The principal *MYOF* finding in the present study was the identification of the rare heterozygous p.Arg1590Gln variant in a 14-year-old girl with a clinical phenotype consistent with HAE-nC1INH and a positive family history. *MYOF* has previously been implicated in HAE-nC1INH, but the evidence supporting individual *MYOF* variants remains limited, and the contribution of *MYOF* variation to disease pathogenesis is not yet fully defined [14,15].

The p.Arg1590Gln variant was evaluated using population data, evolutionary conservation, structural modelling, protein-stability prediction, and multiple computational pathogenicity algorithms. Arg1590 is highly conserved, and the substitution was consistently predicted to alter local molecular interactions and reduce protein stability. These concordant computational findings provide supporting evidence for potential functional relevance and justify prioritization of p.Arg1590Gln for further investigation. However, computational concordance does not constitute independent evidence of pathogenicity and cannot establish a causal relationship with HAE-nC1INH.

Accordingly, under the ACMG/AMP framework applied in this study, *MYOF* p.Arg1590Gln is classified as a VUS, with PP3 applied at Supporting strength; no strong or moderate pathogenic criteria were fulfilled. The variant should therefore be described as a candidate MYOF variant with computational evidence suggesting potential functional relevance, rather than as an established pathogenic or causative variant. The patient’s phenotype and family history provide clinically relevant context but, in the absence of sufficient segregation data, cannot be considered evidence of causality.

The biological plausibility of *MYOF* involvement remains of interest because myoferlin participates in membrane repair, vesicular trafficking, receptor recycling, angiogenesis, and endothelial barrier regulation. Experimental studies of myoferlin biology provide a plausible mechanistic context for investigating MYOF variants in disorders involving vascular permeability; however, such gene-level biological plausibility should not be extrapolated to establish pathogenicity of p.Arg1590Gln. Functional studies specifically evaluating the effect of p.Arg1590Gln on myoferlin expression, stability, membrane trafficking, and endothelial permeability, together with extended familial segregation analysis and identification of additional unrelated carriers with compatible phenotypes, will be required before its disease relevance can be reclassified.

### 3.3. F12 p.Ala207Pro from Distinction Between Variant Detection, Association, and Causality

Pathogenic variants in *F12* represent the best-characterized genetic cause of HAE-nC1INH [2,19]. However, this established gene–disease relationship should not be extrapolated to all *F12* variants. The best-characterized pathogenic HAE-associated *F12* variants cluster in a specific region of the gene and are supported by mechanistic evidence, whereas the clinical significance of variants in other regions requires independent evaluation.

The p.Ala207Pro variant identified in two unrelated patients in the present cohort differs from these established pathogenic *F12* variants. Computational analyses suggested possible alterations in local molecular interactions, protein stability, and pre-mRNA splicing; however, the predictions were discordant across algorithms. More importantly, these computational observations do not demonstrate increased FXII activation, enhanced kallikrein–kinin pathway activity, or increased bradykinin generation.

Population frequency and existing clinical classifications must also be considered when evaluating this variant. Its occurrence at an appreciable frequency in population databases is not compatible with interpretation as a highly penetrant, independently causative allele for a rare Mendelian form of HAE without substantial additional evidence. The identification of p.Ala207Pro in affected individuals in this cohort therefore represents an observation of co-occurrence rather than evidence of causality. In the absence of an appropriately matched control group, the present study also cannot establish enrichment or a genetic association with HAE-nC1INH.

Accordingly, p.Ala207Pro should not be presented as an established HAE-causing variant on the basis of the present findings. The computational results are best regarded as hypothesis-generating observations concerning possible molecular effects. Functional studies directly assessing FXII activation, kallikrein activity, and bradykinin generation, together with comprehensive segregation analysis and appropriately powered case–control studies, would be required to determine whether p.Ala207Pro has any independent or context-dependent relevance to the HAE-nC1INH phenotype.

### 3.4. HS3ST6 Variants as Candidate Susceptibility or Possible Modifier Variants

Four *HS3ST6* missense variants were identified in five unrelated patients. *HS3ST6* encodes heparan sulfate 3-O-sulfotransferase 6, and the known biological functions of heparan sulfate in endothelial signaling, extracellular matrix organization, and growth-factor interactions provide a plausible context for investigating *HS3ST6* variation in disorders of vascular permeability [17]. However, biological plausibility at the gene level does not establish a disease-modifying or causal effect for the individual variants identified in this cohort.

Among these variants, p.Pro199Leu showed the strongest computational evidence of potential functional relevance, including evolutionary conservation, predicted reduction in protein stability, and concordant deleterious predictions from several algorithms. These findings support prioritization of p.Pro199Leu as a candidate variant for further investigation, but they are insufficient to establish pathogenicity or a causal relationship with HAE-nC1INH in the absence of adequate segregation and functional evidence. The evidence supporting p.Ala163Val, p.Ala192Thr, and p.Leu316Val was more limited. Their population frequencies and neutral or modest predicted functional effects do not support interpretation as highly penetrant, independently causative variants. They are therefore best described as candidate susceptibility variants or possible/putative modifiers. Importantly, these terms describe hypotheses rather than established biological roles: the present study was not designed or powered to demonstrate effects on disease susceptibility, penetrance, severity, or phenotypic expression.

Thus, none of the *HS3ST6* variants identified here should be interpreted as an established pathogenic variant or established genetic modifier on the basis of the present data. Larger case–control cohorts, comprehensive family segregation analyses, and functional studies assessing HS3ST6 enzymatic activity and heparan sulfate sulfation will be required to determine whether any of these variants contribute to HAE-nC1INH susceptibility or phenotype.

### 3.5. Limitations

Several limitations should be considered when interpreting our findings. First, the relatively small cohort reflects the rarity of pediatric HAE-nC1INH but limits the statistical power of the study, particularly for genotype–phenotype analyses. The small number of patients carrying individual variants precluded robust assessment of associations with specific clinical manifestations or disease severity. This was further constrained by the absence of a standardized severity measure across the entire cohort and by incomplete family segregation data. Accordingly, the coexistence of a variant with a particular clinical phenotype should not be interpreted as evidence of a causal or modifying effect.

The absence of an unrelated healthy control group is another important limitation. Variant frequencies were evaluated using population reference databases rather than an appropriately matched control population, preventing direct assessment of whether the identified variants are enriched in children with HAE-nC1INH. This is particularly relevant to the common *F12* p.Ala207Pro variant and several *HS3ST6* variants, whose occurrence in affected individuals alone does not establish disease association or causality. Population allele frequencies may also differ across ancestry groups, further complicating interpretation in the absence of ancestry-matched controls. Appropriately designed case–control studies will therefore be required to determine whether any of these variants are enriched in HAE-nC1INH.

Referral and selection bias should also be considered, as the cohort comprised children selected on the basis of a clinical phenotype suggestive of HAE-nC1INH and evaluated at specialized centers. Together with the small sample size and clinical selection of the cohort, this limits the generalizability of our observations to pediatric populations with different genetic backgrounds, clinical characteristics, or referral patterns. Family segregation analysis was also incomplete, particularly for the *MYOF* and *F12* variants. For *MYOF* p.Arg1590Gln, affected relatives were reported in the family history, but informative genotyping of these relatives was unavailable; for *F12* p.Ala207Pro, only limited family testing was available. Consequently, the available data were insufficient to establish cosegregation with disease, assess penetrance systematically, or determine the occurrence of unaffected carriers. The absence of informative segregation evidence was therefore considered when interpreting variant pathogenicity and precluded the use of segregation as evidence supporting causality.

A further limitation is the reliance on computational methods for functional interpretation. Analyses of evolutionary conservation, protein stability, structural effects, splice sites, and predicted functional impact are valuable for variant prioritization but remain hypothesis-generating and cannot establish biological function or disease causality. No experimental studies were performed to determine the effects of *MYOF* p.Arg1590Gln on myoferlin expression, stability, or endothelial function; the effect of *F12* p.Ala207Pro on FXII activation and downstream contact-system activity; or the effects of the *HS3ST6* variants on enzymatic activity and heparan sulfate sulfation. The proposed functional consequences of these variants should therefore be regarded as provisional and require experimental validation.

The technical limitations of WES should also be acknowledged. Although WES provides broad coverage of protein-coding regions, it does not reliably detect all potentially relevant regulatory or deep-intronic variants, structural rearrangements, or certain copy-number changes. A negative WES result therefore does not exclude an underlying genetic contribution to the phenotype. More comprehensive genomic approaches may be required to identify disease-associated alterations outside the regions or variant classes reliably captured by WES.

Interpretation of variants with appreciable population frequencies requires particular caution. Population frequency, ancestry, segregation, clinical phenotype, and functional evidence should be considered collectively, and relatively common variants should not be regarded as independently causative in the absence of additional supporting evidence. This consideration is especially important for *F12* p.Ala207Pro and several *HS3ST6* variants. Likewise, the proposed contribution of *HS3ST6* variants to disease susceptibility or phenotypic variability remains hypothetical and cannot be established from the present cohort.

Larger multicenter studies incorporating appropriately matched control populations, standardized clinical phenotyping, comprehensive family segregation analyses, consistent and more comprehensive genomic approaches, and gene- and variant-specific functional experiments will be required to clarify the biological and clinical significance of these findings. Despite these limitations, the present study provides additional data on the genetic spectrum of pediatric HAE-nC1INH and illustrates the value of integrated bioinformatic analysis for variant prioritization and hypothesis generation when functional evidence is limited.

Finally, the terminology used throughout this study reflects the strength of the available evidence. Established pathogenic variants refer to variants supported by sufficient genetic, clinical, and/or functional evidence for disease causality; previously reported variants with clinical or functional support are distinguished from newly identified variants when the available evidence remains insufficient for definitive pathogenic classification; candidate variants are variants prioritized for further investigation on the basis of genetic, clinical, population, and/or computational evidence; VUS are designated according to ACMG/AMP classification; and candidate susceptibility or putative modifier variants denote variants for which a possible contribution to disease risk, penetrance, or phenotypic expression is hypothesized but not established. Computational predictions are considered supporting, hypothesis-generating evidence within this framework and are not used independently to infer pathogenicity, causality, or a modifying effect.

## 4. Materials and Methods

### 4.1. Study Cohort

**Figure 4:**
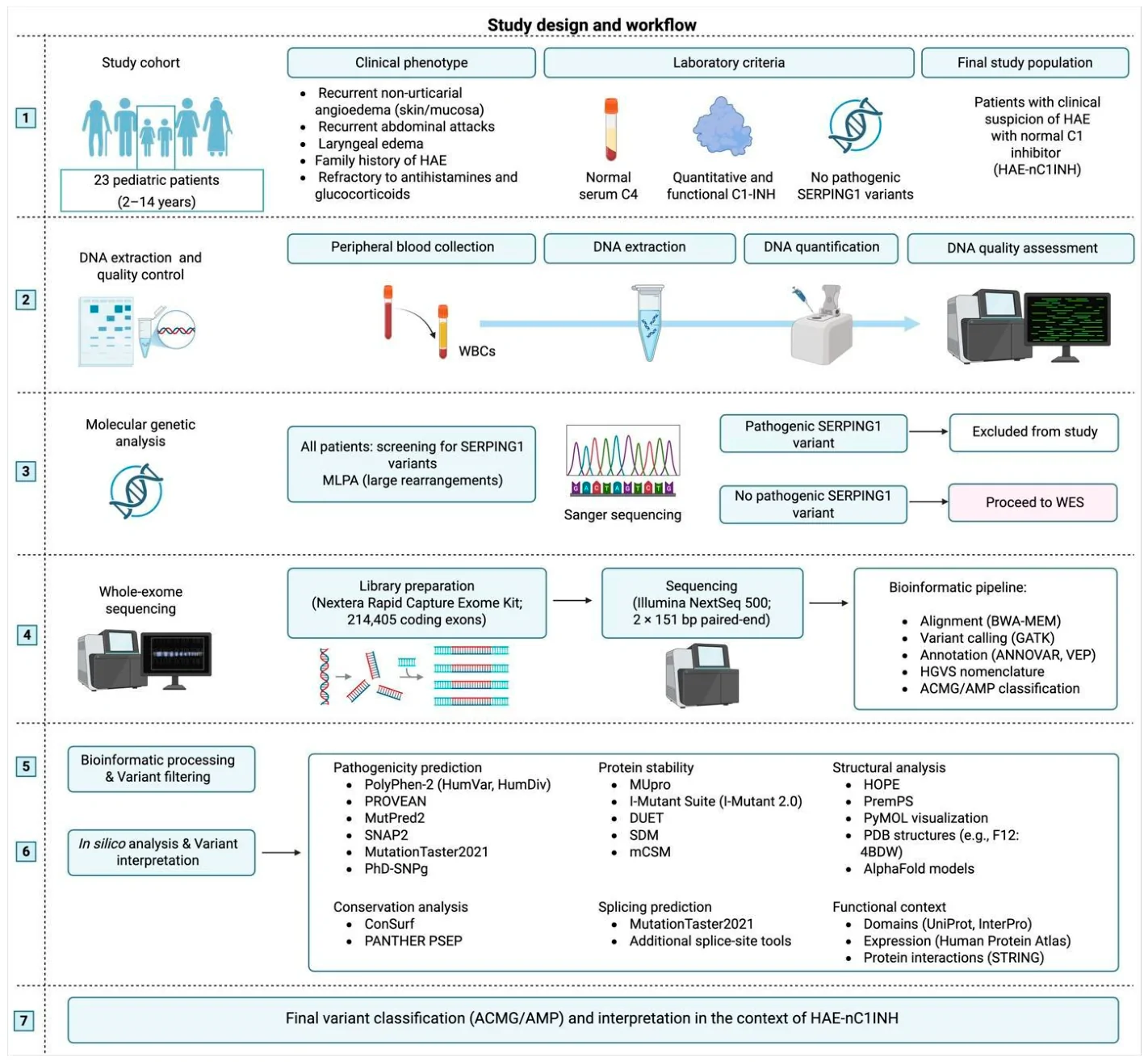
Study design and workflow for molecular genetic investigation of HAE-nC1INH. Overview of the study design and analytical workflow. Twenty-three pediatric patients with recurrent non-urticarial angioedema, recurrent abdominal attacks, laryngeal edema, and/or a positive family history of hereditary angioedema, who were refractory to antihistamines and systemic glucocorticoids, were enrolled. Patients with normal serum C4, normal quantitative and functional C1-INH levels, and no pathogenic *SERPING1* variants were classified as having clinically suspected HAE with HAE-nC1INH. Genomic DNA was extracted from peripheral blood leukocytes, followed by DNA quantification and quality assessment. All samples were initially screened for *SERPING1* variants using multiplex ligation-dependent probe amplification and Sanger sequencing. Patients without pathogenic *SERPING1* variants subsequently underwent WES. Sequencing data were processed using a standardized bioinformatic pipeline including sequence alignment, variant calling, annotation, and ACMG/AMP classification. Candidate variants were further evaluated using an integrated in silico framework incorporating pathogenicity prediction, evolutionary conservation analysis, protein stability assessment, structural modelling, splice-site prediction, functional annotation, protein–protein interaction analysis, and population frequency data. Final variant interpretation was performed by integrating molecular, computational, and clinical evidence according to ACMG/AMP recommendations.

The study included 23 patients aged 2 to 14 years with a clinical picture consistent with HAE (inclusion criteria are presented in Figure 4). Clinical evaluation was performed according to contemporary international recommendations for the diagnosis of HAE. Eligibility criteria comprised a clinical phenotype consistent with HAE, including recurrent non-urticarial angioedema affecting the skin and/or mucosal tissues, recurrent unexplained abdominal attacks, laryngeal edema, and/or a positive family history of HAE, together with normal serum C4 concentrations, normal quantitative and functional C1-INH levels determined by immunoturbidimetry, and the absence of pathogenic *SERPING1* variants confirmed by Sanger sequencing. All participants underwent standardized clinical assessment, laboratory investigations, and molecular genetic analysis. Patients with quantitative or functional C1-INH deficiency or pathogenic *SERPING1* variants were excluded. The final study population therefore comprised patients with HAE-nC1INH.

### 4.2. Ethics Statement

The study protocol was approved by the Interuniversity Ethics Committee of I.M. Sechenov First Moscow State Medical University (Protocol No. 04-25, approved on 20 February 2025) and by the Local Ethics Committee of the Saint Petersburg Pasteur Research Institute of Epidemiology and Microbiology (Protocol No. 17, approved on 25 September 2023). Both approvals covered the clinical and genetic components of the study. All procedures involving human participants were conducted in accordance with the principles of the Declaration of Helsinki [25]. Written informed consent for participation and genetic testing was obtained from the parents or legal guardians of all participants, and age-appropriate assent was obtained from the children when applicable.

### 4.3. DNA Extraction and Molecular Genetic Analysis

Genomic DNA was isolated from peripheral blood leukocytes using a modified phenol–chloroform extraction protocol based on the method of Sambrook et al. [26]. Whole blood was collected by venipuncture into EDTA-containing vacuum tubes (BD Vacutainer, Franklin Lakes, NJ, USA). DNA concentration was quantified using a Qubit 2.0 fluorometer with the Qubit dsDNA High-Sensitivity Assay Kit (Thermo Fisher Scientific, Waltham, MA, USA), whereas DNA integrity and quality were evaluated using an Agilent 2100 Bioanalyzer (Agilent Technologies, Santa Clara, CA, USA). All 23 patients were initially screened for pathogenic variants in *SERPING1*. Large genomic rearrangements were assessed by multiplex ligation-dependent probe amplification, followed by Sanger sequencing of the coding exons and exon–intron boundaries. No pathogenic *SERPING1* variants were identified in the 23 patients included in the study. All 23 patients subsequently underwent WES. No separate targeted sequencing panel of HAE-associated genes was performed. The *MYOF*, *F12*, and *HS3ST6* variants reported in this study were identified by WES. Sanger sequencing was used for the initial *SERPING1* analysis and was not performed as a separate confirmatory procedure for the WES-identified variants.

### 4.4. Whole-Exome Sequencing

WES was performed in all 23 patients included in the final cohort on an Illumina NextSeq 500 instrument using 2 × 151 bp paired-end reads, achieving a mean target coverage of 70–100×. Exome libraries were generated using the Nextera Rapid Capture Exome Kit (Illumina, San Diego, CA, USA), targeting 214,405 coding exons, according to the manufacturer’s protocol. Raw sequencing data were generated in FASTQ format. All samples included in the final analysis were processed using the same reference genome and variant-calling pipeline.

### 4.5. Bioinformatic Analysis and Variant Prioritization

Raw sequencing data were processed using the Illumina DRAGEN Bio-IT Platform. Following adapter removal and automated quality filtering, sequencing reads were aligned to the GRCh37/hg19 human reference genome, using the corresponding exome target-region BED file. Single-nucleotide variants (SNV) and small insertions/deletions were identified using the integrated DRAGEN variant-calling pipeline and annotated against dbSNP. The resulting variants were further evaluated using Variant Interpreter (Illumina, https://www.illumina.com/informatics/biological-interpretation/variant-analysis.html, accessed on 5 March 2024), and additional population-frequency and ClinVar clinical-significance annotations were incorporated using custom Python scripts. All variants presented in the Results were derived from this GRCh37/hg19-based DRAGEN workflow, and all samples were processed using the same pipeline. No coordinate conversion between GRCh37/hg19 and GRCh38/hg38 was performed for the primary analysis or variant reporting. Variant filtering and prioritization incorporated sequencing quality, read depth, genotype, predicted molecular consequence, population frequency, clinical annotation, and biological relevance to the HAE-nC1INH phenotype. In the primary DRAGEN-derived dataset, QUAL ≥ 30 and DP ≥ 4 were used for routine variant screening. The relatively permissive depth threshold was intended as an initial screening criterion rather than as independent evidence of a reliable germline call. Potentially relevant heterozygous variants could therefore be retained for further review at lower quality when supported by additional genetic, clinical, or functional annotations. Candidate selection prioritized homozygous nonsynonymous variants and heterozygous variants of potential clinical relevance, as well as variants predicted to result in missense, frameshift, stop-gain, stop-loss, start-loss, or splice-region consequences.

Genotype quality, allele balance, mapping quality, and strand-bias metrics were not subjected to additional fixed post hoc numerical thresholds during candidate prioritization beyond the quality-control procedures implemented in the primary sequencing and DRAGEN workflow; therefore, retrospective cutoffs for these parameters were not introduced. Similarly, population allele frequency was considered as part of an integrated variant assessment rather than used as a single absolute exclusion criterion. Candidate variants were prioritized by integrating population frequency, predicted consequence, available ClinVar evidence, genotype, and biological relevance of the affected gene. Variants retained for interpretation were described according to HGVS nomenclature and evaluated in accordance with ACMG/AMP recommendations.

### 4.6. In Silico Analysis of Sequence Variants

WES data were evaluated for variants in genes associated with HAE-nC1INH, including *F12*, *PLG*, *ANGPT1*, *KNG1*, *MYOF*, and *HS3ST6*, as well as other potentially relevant coding variants identified across the exome.

Rare coding variants were prioritized according to allele frequency, predicted functional consequences, inheritance pattern, and biological relevance to the kallikrein–kinin pathway or endothelial homeostasis. Variant nomenclature followed the recommendations of HGVS, and clinical interpretation was performed in accordance with the American College of Medical Genetics and Genomics/Association for Molecular Pathology (ACMG/AMP) guidelines.

The pathogenic potential of identified missense variants was evaluated using multiple complementary computational approaches [27]. Functional effects of amino acid substitutions were predicted using PolyPhen-2 (HumVar and HumDiv models, http://genetics.bwh.harvard.edu/pph2/, accessed on 5 March 2024) [27], PROVEAN (https://www.jcvi.org/research/provean, accessed on 5 March 2024) [28], MutPred2 (http://mutpred2.mutdb.org/index.html, accessed on 5 March 2024) [29], SNAP2 (https://github.com/Rostlab/SNAP2, accessed on 5 March 2024) [30], MutationTaster2021 (http://www.mutationtaster.org, accessed on 5 March 2024) [31], and PhD-SNPg (https://snps.biofold.org/phd-snpg/, accessed on 5 March 2024) [32]. These tools provided predictions of pathogenicity together with probability scores, functional impact, and evolutionary constraint metrics where available. PolyPhen-2 predictions were interpreted using both the HumVar and HumDiv training models to evaluate the potential effects of amino acid substitutions on protein structure and function. Protein stability was assessed using MUpro (http://mupro.proteomics.ics.uci.edu/, accessed on 7 March 2024) [33,34], I-Mutant Suite (I-Mutant2.0) (http://gpcr.biocomp.unibo.it/~emidio/I-Mutant2.0/dbMut.html, accessed on 7 March 2024) [35], DUET (http://biosig.unimelb.edu.au/duet/, accessed on 7 March 2024) [36] with assessment of SDM, and mCSM [34,37]. Changes in protein stability were evaluated based on predicted Gibbs free energy differences (ΔΔG) following single amino acid substitutions, together with confidence or reliability scores provided by the individual algorithms. Structural consequences of missense variants were investigated using HOPE (https://www3.cmbi.umcn.nl/hope, accessed on 7 March 2024) [38] and PremPS (https://lilab.jysw.suda.edu.cn/research/PremPS/, accessed on 7 March 2024) [39]. Three-dimensional protein structures were visualized and analyzed using PyMOL version 2.4.0, and, when available, experimentally determined Protein Data Bank (PDB) structures (e.g., FXII, PDB ID: 4BDW) were used as structural templates. Structural interpretations were additionally supported by AlphaFold protein models when experimentally resolved structures were unavailable [40].

Evolutionary conservation of affected amino acid residues was assessed using both ConSurf (https://consurf.tau.ac.il/, accessed on 10 March 2024) [41] and the PANTHER Position-Specific Evolutionary Preservation (PSEP) algorithm (https://pantherdb.org/tools/csnpScoreForm.jsp, accessed on 10 March 2024) [42,43], which estimates residue conservation over evolutionary time and predicts the likelihood that amino acid substitutions affect protein function. Potential effects on pre-mRNA splicing were evaluated using MutationTaster2021 together with additional splice-site prediction algorithms where appropriate.

Protein functional domains were annotated using UniProt (https://www.uniprot.org/, accessed on 10 March 2024) [44] and InterPro (https://www.ebi.ac.uk/interpro/, accessed on 10 March 2024) [45], whereas tissue-specific gene expression profiles were examined using the Human Protein Atlas. Protein–protein interaction networks were explored using STRING (https://string-db.org/, accessed on 10 March 2024) [46] to investigate the functional relationships of candidate genes within pathways regulating vascular permeability and the kallikrein–kinin system.

Population allele frequencies were obtained from the Genome Aggregation Database (gnomAD) and NCBI dbSNP, while previously reported clinical interpretations were retrieved from ClinVar. Computational predictions were interpreted as supporting evidence and integrated with clinical phenotype, family segregation, population allele frequencies, structural modeling, and molecular genetic findings during final variant classification.

## 5. Conclusions

HAE-nC1INH remains a genetically heterogeneous and diagnostically challenging disorder, particularly in children, for whom validated biochemical biomarkers are lacking. Molecular genetic testing therefore represents an important component of the diagnostic evaluation once C1-inhibitor deficiency and other relevant causes of recurrent angioedema have been excluded [1].

In this pediatric cohort, variants were identified in *MYOF*, *F12*, and *HS3ST6*, but their clinical significance varied considerably. *MYOF* p.Arg1590Gln was classified as a VUS according to ACMG/AMP criteria and should be considered a candidate variant rather than an established cause of disease. *F12* p.Ala207Pro was identified in two affected patients; however, its population frequency, discordant computational predictions, and absence of functional evidence do not support a causal role on the basis of the present data. Among the *HS3ST6* variants, p.Pro199Leu showed the strongest computational evidence and warrants further investigation, whereas p.Ala163Val, p.Ala192Thr, and p.Leu316Val should be regarded as candidate susceptibility or putative modifier variants whose clinical effects remain unestablished.

These findings underscore the distinction between variant identification, computational prioritization and evidence of disease causality. In silico analyses can prioritize candidates and generate experimentally testable hypotheses, but cannot independently establish pathogenicity or modifier effects [7]. Definitive interpretation will require larger, appropriately controlled cohorts, family segregation analyses and variant-specific functional studies [1].

A further limitation is the absence of standardized individual-level phenotyping across all variant-positive patients, which precluded robust genotype–phenotype analysis. Prospective studies integrating systematic assessment of attack characteristics, treatment response, family history and laboratory parameters with genomic and functional data will be important for defining the clinical relevance of candidate variants in pediatric HAE-nC1INH.

## Figures and Tables

**Table 1:** Demographic and clinical characteristics of the study cohort.

Parameter	Value
Demographic characteristics	
Number of patients, *n*	23
Male, *n* (%)	9 (39.1)
Female, *n* (%)	14 (60.9)
Median age at evaluation, years [range]	8 [2–14]
Positive family history of angioedema, *n* (%)	8 (34.8)
Disease onset	
Median age at onset, years [range]	6 [1–11]
Onset within the first decade of life, *n* (%)	16 (69.6)
Clinical manifestations at disease onset	
Subcutaneous angioedema, *n* (%)	13 (56.5)
Abdominal attacks, *n* (%)	10 (43.5)
└ Isolated abdominal attacks	7 (30.4)
└ Abdominal pain with peripheral edema	3 (13.0)
Upper airway edema requiring emergency medical care, *n* (%)	1 (4.3)
Molecular genetic findings	
Patients carrying variants prioritized for further evaluation, *n* (%)	8 (34.8)

└ indicates a subcategory of the preceding attack type.

**Table 2:** Summary and population-frequency annotation of genetic variants identified and evaluated in the study cohort.

Gene/Protein Variant	GRCh37/hg19 Genomic HGVS (NCBI dbSNP Build 157/NHLBI TOPMed, Access 11 September 2026)	RefSeq Transcript/cDNA HGVS	Allele Frequency Based on TOPMed Global/dbSNP ID
*MYOF* p.Arg1590Gln	NC_000010.10:g.95093020C>T (accessed on 11 September 2026 https://www.ncbi.nlm.nih.gov/snp/rs201619869#hgvs_tab)	NM_013451.4:c.4769G>A	0.000026 (Global, multi-ancestry)/ rs201619869
*F12* p.Ala207Pro	NC_000005.9:g.176831826C>G (accessed on 11 September 2026 https://www.ncbi.nlm.nih.gov/snp/rs17876030#hgvs_tab)	NM_000505.4:c.619G>C	0.954433 (Global, multi-ancestry)/ rs17876030
*HS3ST6* p.Ala163Val	NC_000016.9:g.1962132G>A (accessed on 11 September 2026 https://www.ncbi.nlm.nih.gov/snp/rs8055325#hgvs_tab)	NM_001009606.4:c.488C>T	0.386592 (Global, multi-ancestry)/ rs8055325
*HS3ST6* p.Ala192Thr	NC_000016.9:g.1962046C>T (accessed on 11 September 2026 https://www.ncbi.nlm.nih.gov/snp/rs1742399#hgvs_tab)	NM_001009606.4:c.574G>A	0.324912 (Global, multi-ancestry)/ rs1742399
*HS3ST6* p.Pro199Leu	NC_000016.9:g.1962024G>A (accessed on 11 September 2026 https://www.ncbi.nlm.nih.gov/snp/rs189679573#hgvs_tab)	NM_001009606.4:c.596C>T	0.001111 (Global, multi-ancestry)/ rs189679573
*HS3ST6* p.Leu316Val	NC_000016.9:g.1961674G>C (accessed on 11 September 2026 https://www.ncbi.nlm.nih.gov/snp/rs337285#hgvs_tab)	NM_001009606.4:c.946C>G	0.550822 (Global, multi-ancestry)/ rs337285

**Table 3:** Comprehensive evidence and ACMG/AMP interpretation of the MYOF p.Arg1590Gln variant.

Parameter	Evidence/Interpretation
Gene	*MYOF*
dbSNP ID	rs201619869
Variant type/consequence	Single-nucleotide variant; missense variant *MYOF* rs201619869 (p.Arg1590Gln)
Genome assembly used for primary reporting	GRCh37.p13 (hg19)
Genomic position (GRCh37)	chr10:95093020 C>T
Genomic HGVS (GRCh37)	NC_000010.10:g.95093020C>T
Equivalent genomic position (GRCh38)	chr10:93333263 C>T (GRCh38.p14)
Reference transcript	NM_013451
Transcript version	NM_013451.4
Transcript designation	*MYOF* transcript variant 1; RefSeq Select
cDNA HGVS	NM_013451.4:c.4769G>A
mRNA sequence context	5′-AAGTCATTGAAGACC[G]AGATCACTACATTC-3′
mRNA nucleotide substitution	G>A
Protein reference	NP_038479.1
Protein designation	Myoferlin isoform a
Protein HGVS	NP_038479.1:p.Arg1590Gln (R1590Q)
Zygosity in the index patient	Heterozygous (0/1)
Sequencing depth at variant position	6× total depth
Population allele frequency—gnomAD exomes	T = 0.0000535 (75/1,401,478)
Population allele frequency—TOPMed	T = 0.000026 (7/264,690)
Population allele frequency—ALFA global	T = 0.000060 (13/215,332)
ALFA European	T = 0.000050
ALFA African	T = 0.0001
ALFA Asian	T = 0.0002
ALFA Latin American 1	T = 0.001
ALFA Latin American 2	T = 0.000
ALFA South Asian	T = 0.000
ExAC global	T = 0.000058
ExAC European	T = 0.00005
Population-frequency interpretation	Rare but present in population databases.
ClinVar status/classification	Not reported in ClinVar
Published evidence	The same variant has previously been reported in a female patient with clinical manifestations of HAE and normal C1-INH in the heterozygous state. The reported evidence for potential functional relevance was predominantly computational.
Segregation evidence	No sufficient familial cosegregation evidence available to support pathogenicity.
De novo status	Not established.
Case–control/enrichment evidence	No statistically supported enrichment in HAE-nC1INH cases compared with controls has been demonstrated.
Experimental functional evidence	Not available. No well-established functional assay specific to p.Arg1590Gln has demonstrated an effect relevant to HAE-nC1INH.
PolyPhen-2 HumVar	0.953
PolyPhen-2 HumDiv	1.000
PhD-SNPg	0.967
Other computational evidence	Evolutionary conservation, structural modeling, and protein-stability analyses suggest possible effects on myoferlin structure/function. These findings represent computational rather than experimental evidence.
PVS1	Not met—p.Arg1590Gln is a missense substitution rather than an expected loss-of-function variant.
PS1	Not met—no established pathogenic variant resulting in the same amino-acid substitution has been demonstrated.
PS2	Not met—de novo occurrence has not been established.
PS3	Not met—no well-established functional assay demonstrates a damaging effect of p.Arg1590Gln.
PS4	Not met—statistically significant enrichment in affected individuals compared with controls has not been demonstrated.
PM1	Not met—available domain/conservation evidence is insufficient to establish Arg1590 as a mutational hotspot or critical functional region with little benign variation.
PM2	Not assigned—the variant is rare but present in population databases (gnomAD exomes AF = 5.35 × 10^−5^), and no validated MYOF/HAE-nC1INH-specific frequency threshold is available to justify this criterion.
PM5	Not met—no established pathogenic missense variant affecting the same amino-acid residue has been demonstrated.
PP1	Not met—informative cosegregation with HAE-nC1INH has not been demonstrated.
PP2	Not met—*MYOF* missense variation has not been established as a common disease mechanism for HAE-nC1INH.
PP3	Met—Supporting—multiple computational analyses support a possible deleterious effect on protein structure/function.
PP4	Not met—HAE-nC1INH is genetically heterogeneous, and the phenotype is not sufficiently specific for a *MYOF*-related etiology.
BA1	Not met—population frequency is below a stand-alone benign threshold.
BS1	Not assigned—no validated maximum credible allele-frequency threshold for a *MYOF*-related HAE-nC1INH mechanism is available to establish that the observed frequency is incompatible with disease.
BS2–BS4	Not met—adequate benign observation, segregation, or functional evidence is unavailable.
BP criteria	Not met—no applicable benign supporting criterion can be assigned from the currently available evidence.
ACMG/AMP criteria applied	PP3 (Supporting)
Final ACMG/AMP classification	Variant of Uncertain Significance (VUS)
Evidence level	Predominantly computational/supporting evidence; no direct functional or sufficient segregation evidence.
Interpretation in the present study	Candidate variant with computational evidence suggesting potential functional relevance.
Causality statement	The currently available evidence is insufficient to establish MYOF p.Arg1590Gln as causative for HAE-nC1INH.

**Table 4:** Comprehensive evidence and ACMG/AMP interpretation of the *F12* p.Ala207Pro variant.

Parameter	Evidence/Interpretation
Gene	*F12*
dbSNP ID	rs17876030
Variant type/consequence	Single-nucleotide variant (SNV); coding missense variant
Genome assembly used for primary reporting	GRCh37.p13 (hg19)
Genomic position (GRCh37)	chr5:176831826 C>G
Genomic HGVS (GRCh37)	NC_000005.9:g.176831826C>G
Equivalent genomic position (GRCh38)	chr5:177404825 C>G
Reference transcript	NM_000505
Transcript version	NM_000505.4
Transcript designation	MANE Select; RefSeq Select
cDNA HGVS	NM_000505.4:c.619G>C
mRNA sequence context	5′-GTGGGCTACACCGGA[G]CCTTCTGCGACGTG-3′
mRNA nucleotide substitution	G>C
Protein reference	NP_000496.2
Protein designation	Coagulation factor XII preproprotein
Protein HGVS	NP_000496.2:p.Ala207Pro
Zygosity in the index patient	Homozygous; homozygosity was identified in two unrelated affected patients in the present cohort.
Sequencing depth at variant position	Not specifically reported for this variant. The bioinformatic pipeline retained variants with DP > 4; therefore, the exact variant-level DP should be retrieved from the original VCF rather than inferred from the filtering threshold.
Population allele frequency—gnomAD exomes	~0.9653 for the allele represented by the p.Ala207Pro ClinVar record
Population allele frequency—TOPMed	~0.9544 for the allele represented by the p.Ala207Pro ClinVar record
Population-frequency interpretation	The allele corresponding to p.Ala207Pro is very common in contemporary population datasets. Its frequency is incompatible with interpretation as a highly penetrant, independently causative allele for a rare Mendelian form of HAE. Genomic and transcript alleles must be interpreted carefully because *F12* is encoded on the reverse strand.
ClinVar status/classification	Benign. Current ClinVar records classify NM_000505.4:c.619G>C (p.Ala207Pro) as benign; benign classifications include *F12*-related conditions.
Published evidence	Previously reported in population and clinical datasets, including individuals with angioedema. Observation in affected individuals does not itself demonstrate pathogenicity, disease association, or causality.
Segregation evidence	In one family, the affected index patient was homozygous and the mother was heterozygous. The available segregation data in the present manuscript are insufficient to demonstrate cosegregation with disease.
De novo status	Not de novo in the family with available parental testing; maternal inheritance of one allele was demonstrated.
Case–control/enrichment evidence	No statistically robust enrichment supporting association with HAE-nC1INH has been demonstrated. The present study lacks an unrelated matched healthy control group and therefore cannot establish variant enrichment.
Experimental functional evidence	None in the present study. No variant-specific assays of FXII activation, kallikrein activity, downstream contact-system activity, or bradykinin generation were performed.
PolyPhen-2 HumVar	0.953; probably damaging
PolyPhen-2 HumDiv	1.000; probably damaging
PhD-SNPg	0.967; predicted pathogenic
Other computational evidence	Discordant overall. Structural modelling predicted altered local interactions; stability algorithms predicted reduced protein stability (ΔΔG approximately −0.54 to −1.63 kcal/mol); MutationTaster2021 predicted increased donor splice-site strength (0.56→0.77). However, SNAP2 and PROVEAN predicted a neutral functional impact. Thus, the computational evidence is hypothesis-generating rather than evidence of causality.
PVS1	Not met—p.Ala207Pro is a missense variant; the PVS1 loss-of-function criterion is not applicable
PS1	Not met—no established pathogenic variant producing the same amino-acid substitution was identified as evidence for this criterion
PS2	Not met—de novo occurrence was not demonstrated
PS3	Not met—no well-established variant-specific functional study demonstrates a pathogenic effect relevant to HAE
PS4	Not met—no statistically significant enrichment in affected individuals compared with controls has been demonstrated
PM1	Not met—Ala207 is not within the region containing the best-characterized HAE-associated *F12* variants, and no established mutational hotspot applicable to this residue has been demonstrated. The manuscript notes that established HAE-associated *F12* variants predominantly cluster elsewhere.
PM2	Not met—the variant/allele is common in population databases
PM5	Not met—no established pathogenic missense substitution at Ala207 supports application of this criterion
PP1	Not met—available family data are insufficient to demonstrate cosegregation with disease
PP2	Not met—insufficient evidence for application of this criterion
PP3	Not applied—although several computational tools predicted a damaging effect, predictions were discordant, with SNAP2 and PROVEAN predicting a neutral effect.
PP4	Not met—the clinical phenotype is not sufficiently specific to p.Ala207Pro to provide variant-level pathogenic evidence
BA1	Met (Stand-alone)—the population frequency of the allele corresponding to p.Ala207Pro is far above the ACMG/AMP stand-alone benign frequency threshold and is incompatible with a rare highly penetrant Mendelian HAE-causing allele
BS1	Consistent with benign population evidence but not independently required once BA1 is applied; the observed population frequency substantially exceeds that expected for a rare highly penetrant HAE-causing allele
BS2–BS4	Not formally applied—additional benign criteria are not required for the present classification once BA1 is applied; available evidence was not used to assign these criteria independently
BP criteria	Not applied—no additional BP criterion is required for the final classification; discordant computational predictions were not used as independent benign evidence
ACMG/AMP criteria applied	BA1 (Stand-alone)
Final ACMG/AMP classification	Benign (B)
Evidence level	Strong population evidence against a highly penetrant Mendelian disease-causing role; classification is concordant with current ClinVar benign interpretation. Computational findings provide only hypothesis-generating evidence and do not override the population evidence.
Interpretation in the present study	Common benign variant and should not be interpreted as an independently causative HAE-nC1INH variant. Its homozygous occurrence in two affected patients represents co-occurrence rather than evidence of disease causality. The predicted structural, stability, and splice effects remain computational observations without variant-specific functional confirmation.
Causality statement	The available evidence does not support a causal relationship between *F12* p.Ala207Pro and HAE-nC1INH. Any potential context-dependent susceptibility or modifier effect remains unproven and would require independent case–control, segregation, and functional evidence.

## Data Availability

The data generated in this study include genomic information from pediatric participants and are therefore subject to privacy, consent, and ethical restrictions. The informed consent obtained from parents or legal guardians did not include unrestricted public sharing of individual-level genomic data. Accordingly, these data are not publicly available. De-identified data supporting the findings of this study may be made available by the corresponding author upon reasonable request and subject to approval in accordance with applicable ethical and institutional requirements. Where permitted by the scope of informed consent and ethics approvals, appropriately de-identified variant-level information may also be made available through a controlled-access repository.
